# Supplementary material for: Patterns of Intron Gain and Loss in Fungi
Source: PLoS Biol. 2004 Nov 30;2(12):e422. doi: 10.1371/journal.pbio.0020422 (PMC532390; doi:10.1371/journal.pbio.0020422)
Supplement: Table S1 — Also available at http://genes.mit.edu/NielsenEtAl/. (4.3 MB ZIP). [file pbio.0020422.st001.zip › NielsenEtAl/html/908.html]

AN8042.1.NCU01525.1.MG01076.1.FG04324.1


```
 CLUSTAL W (1.82) Multiple Sequence Alignments - Introns Inserted


Sequence 1: NCU01525.1	744 aa
Sequence 2: MG01076.1	758 aa
Sequence 3: FG04324.1	802 aa
Sequence 4: AN8042.1	744 aa
Alignment Length: 869 aa
Number Identitical Residues: 249 aa
Alignment Score (without introns) 15062


MG01076.1 	---------------------------MEEDAVMEGDGPLPVNGDSNGYPNARDIAAGFN
NCU01525.1	--------------------------------MVDNDNDIVMDGQ-DTHPATIDVSATSN
FG04324.1 	---------------------------------MEPNATDIVDHP------MKDLDAPNG
AN8042.1  	MVDDEDIIMGEAGYHSANPNTAIAAGQQTTHGHLNGRSDPYLAPPLLSQPMQMPVVQAGN
          	  ....   ..:.  ::...:: ::..    . ::      :       .    :    .

MG01076.1 	DDQMQLQFGDDED---------GDVQLHLEDDGDNELELYNPRDFPDDLRRR--------
NCU01525.1	GITKTEPNGHDQS---------APANDEKEEKLDDEKD---PFIYPARLKRR--------
FG04324.1 	SYQTRNTSIDDDD---------TASEDPYEADVDPEPQ----DITFDQMRRR--------
AN8042.1  	SQTQESSATTDEARVLSPKIDLNSTVPADAADITPDYQDSLVDEYSDYAEESKEAPGLPL
          	.         *:    :.. .          .   : :           .. ..:.. . 

MG01076.1 	GLLPTGCCYDDRMKLHANADFGAGPHHPEDPSRIEAIFKTFKQAGLVFTGTDSELIDIIA
NCU01525.1	GLLPTGCCYDDRMKLHANADFGPNPHHPEDPSRIEHIMRIFKKEGLVFTGDDEDLKKVIR
FG04324.1 	GLLPTGCCYDDRMKLHMNADFSPNTHHPEDPRRIHEIFKAFKKVGLVYTGPEAELPRIMR
AN8042.1  	ASLPSGLCYDVQMRYHCEVRP-TSDVHPEDPRRIYYIYKELCRAGLV---DDIESTRPLV
          	. **:* *** :*: * :.   ..  ***** **  * : : : ***    : :    : 

MG01076.1 	NKPSSYMWRIPAREATKEEICLVHYAKHYDWVKELST~KPTAQLRRLTKELDKGQTSVYV
NCU01525.1	TDPRRYMWRIPARHATKEEICIVHHPEHFRWVEDLSR~KPTSELRRLSTIMDQGRDSLYV
FG04324.1 	ECPTRYMWRIPARAATREEICLAHSPEHFDWVERLDK~MSTAELRDLTKQYDQGRESLYV
AN8042.1  	ARP---LKRIHARNATEEEISLVHTAAHYAFVESTKD1MSDEELIAL----EHTRDSIYF
          	  *   : ** ** **.***.:.* . *: :*:  .   .  :*  *    :: : *:*.

MG01076.1 	GSLTFEASLISAGGAIETCKSIVSGQIKNGFAVIRPPGHHAEQDSAMGFCIFNNV~PIAA
NCU01525.1	GSMTFEAALISAGGAIETCKSVVVGNVKNAFAVIRPPGHHAEFDAPMGFCLFNNV~PIAA
FG04324.1 	GSMSYPAALLSAGGAIETCKNVVIGQVKNAFAVIRPPGHHAEWDGPMGFCFFNNV~PVAV
AN8042.1  	NNLTFASSLLSVGGAIETCLAVATRKVKNAIAVIRPPGHHAEHDKTMGFCLFNNV2QLKE
          	..::: ::*:*.*******  :.  ::**.:*********** * .****:****  :  

MG01076.1 	KVCQAEYPEICQKVLILDWDVHHG~NGIQNVFYEDPNVLYISLHVYKNGEFYPGKPENPM
NCU01525.1	KICQTEYPEICRKILILDWDVHHG~NGIQNMFYDDPNILYISLHVYMNGSFYPGKPDNPM
FG04324.1 	RVCQQDYPKICRKVLILDWDVHHG~NGVQNIFYNDPNVLYISLHVYQNGIFYPGKPPNSM
AN8042.1  	TNHSR--------------DVHHG1NGIQKAFYDDPNVLYISLHVYQNGSFYPGE-----
          	   .               ***** **:*: **:***:******** ** ****:     

MG01076.1 	TADGSISSCGSGPGLGR~NINIGWDDQGMGDGEYIAAFQKIIMPIAHEFNPDLVII~SAG
NCU01525.1	TPDGSIENCGAGPGLGK~NVNIGWHDQGMGDGEYMAAFQKIVMPIAHEFDPDLVII~SAG
FG04324.1 	TPDGGIEHCGSEAGLGK~NINIGWHDQGMGDGEYMAAFQKIIMPIAKEFDPDLVVI~SAG
AN8042.1  	-KDGDADFCGAGAGEGN2--------QGMGDGDYIYAFHQVVMPIAQEFDPDLVII1ASG
          	  **. . **: .* *.         ******:*: **::::****:**:****:* ::*

MG01076.1 	FDAAAGDQLGGCFVTPPCYAHMTHMLMSLAHGRVAVCLE~GGYNLSAISKSALAVARTLM
NCU01525.1	FDAADGDELGACFVSPACYAHMTHMLMSLANGKVAVCLE~GGYNLAAISKSALAVARTLM
FG04324.1 	FDAADGDELGGCFVSPACYAHMTHMLMSLADGKVAVCLE~GGYNLKAISVSAVAVAKTLM
AN8042.1  	FDAAAGDTLGGCFVTPACYAHMTHMLMTLAQGKVAVCLE0GGYNFRSISKSALAVTKTLM
          	**** ** **.***:*.**********:**.*:****** ****: :** **:**::***

MG01076.1 	GEPPPKMGMASPKINREAARLLAQVQAYQAPYWECMRPGVVDVEEEQRLGSERLHD~VIR
NCU01525.1	GEPPPKMDLP--KINKEAARVLAKVQAYQAPYWECMRPGIVDVQEMQSQGGQRLHD~VVR
FG04324.1 	GEPPPRLELP--KINKEAARILAKVQSLQAPYWECMRPGVVNVPEIQSLNSSRLHD~VIR
AN8042.1  	GNPPDRLSFT--CPSEAAISTIRRVSSIQSDYWKCMYPKAIKNE---GVWTDRLHD1IIR
          	*:** :: :.    .. *   : :*.: *: **:** *  :.         .**** ::*

MG01076.1 	QAQKWKLSKDFGMLPLYIQRENAYKSFENQVLVT~PRIHLARKIMVIIHDA2PELLAQPD
NCU01525.1	SAQRFNLSEKFGMFPLYIQRDVLFKSFENQVLVT~RNASEARKLLVVIHDP2PELHAQPD
FG04324.1 	NAQRQVLQTKHNMIPLYIQRENLYKSYENQVLVT~PGLHEAKKILIIIHDP2PQLLAQPD
AN8042.1  	AYQAKQLYDNYKLTSLYIYRTAISKSFENQVLAR2----------------~PEIMGLPH
          	  *   *  .. : .*** *    **:*****.                   *:: . *.

MG01076.1 	PLDNHLDPHNAWV0NDGVYQYINWAVKEKFGVIDVNVPQYITRPE0DTDAHTRATDEREL
NCU01525.1	PLDNSLQPHDSWI0TDSIPQYIQWAINRKIGVIDINIPQYITHPE0DTESLAPKVDERTQ
FG04324.1 	VIDSTLDPHNAWV0VDGVTDYIDWAIDQKFGVMDINIPAYVTHDE0DSDAHIPSFKEQAL
AN8042.1  	PLTNNLEAHNCWL0ADVMKDYVQWAVGKGYAVIDVNIPKHVTTEP0SSGRFEEEDENR--
          	 : . *:.*:.*:  * : :*::**: .  .*:*:*:* ::*    .:       .::  

MG01076.1 	QRQILEITTFIWDNYLQ~LYDSAEDIFLIGVGAAYLGIKTLLINRE~CK-PRISGVVSFV
NCU01525.1	QAQIQELMCYIWDNYLQ~LYDNVDDIFLMGVGNAYLGVKLLLINRD1VK-SRISGVVNFV
FG04324.1 	TEQIQTLVCYLWDNHLQ~LYD-ADDIFIMGVGNAYLGVKVLLINRD1CK-SRITGVVNFA
AN8042.1  	PTATEELAGYLWDNYID2PNE-ATEIFLVGIGNAFYGVANLLINRE1TLYQRVNGVISFV
          	      :  ::***:::   : . :**::*:* *: *:  *****:     *:.**:.*.

MG01076.1 	TGN-LRPVKSDVDPDLSTWYRD~NSRIYVGSDHACWLDNDLTRKVNKRRFGAVKRSPVTG
NCU01525.1	NGN-LRPVKSDVDTDLSSWYKD~NSRVYVSADHACWADADLSRKVMKRRFGSVIRSNVSG
FG04324.1 	NGT-LRPIKSDIDTDLSSWYKE~NSRVFIAGDHACWADASLTKKVNKRRFGSVVRSPMFG
AN8042.1  	AKDPVRAVASHTQVWLSRWYRD0NSLVFVSKAHGVWKSD---RKATKR-YGKLIQSSREG
          	   .:*.: *. :  ** **:: ** :::.  *. * .    :*. ** :* : :*   *

MG01076.1 	LARMMREHAQDAQAWIRGRVRTPED-GGDTTEEDD~GVRE---------~----------
NCU01525.1	LGRMMNEHFKDVQDWMVDRMVPEEEKLGDEMVEDA~-------------~----------
FG04324.1 	LNKMMAHHAKEAREWILERLEGGSD--ADMTTEEK2GISLLLHVMTSTN2SYCLPSSGMR
AN8042.1  	LSDMLMHHKDEVFQWIEDRIEPESE----ETEEEK~PVKRSPTKAAPKA~A---------
          	*  *: .* .:.  *:  *:   .:       *:           :..  :         

MG01076.1 	~---------------------------------------------
NCU01525.1	~---------------------------------------------
FG04324.1 	2CLQPEPWRSGIQCGAVIARYLSGCLSATRTSACEISDLPAPSKPS
AN8042.1  	~---------------------------------------------
          	
```
